# ICODE: the international COVID-19 thrombosis biomarkers colloquium—novel soluble biomarkers: circulating cell-free nucleic acids and other molecules

**DOI:** 10.1007/s11239-021-02468-6

**Published:** 2021-05-12

**Authors:** Krystin Krauel, Daniel Duerschmied

**Affiliations:** 1 Cardiology and Angiology I, Heart Center, University of Freiburg, Freiburg, Germany; 2 Medical Intensive Care, Medical Center, Faculty of Medicine, University of Freiburg, Freiburg, Germany

## Abstract

This article summarizes the evidence derived from clinical (observational) studies describing novel soluble biomarkers in COVID-19. Our goal was to stimulate further research (preclinical as well as clinical studies) and therefore we discuss potential prognostic value, but also technical details, such as sample preparation. A table provides an overview of the described biomarkers measured in plasma, serum or other (namely bronchoalveolar) fluids.

## Highlights

- Immunothrombosis in COVID-19 is associated with the release of neutrophil-derived molecules, including neutrophil extracellular traps (NETs).
- Myeloperoxidase/DNA complexes have been well established as biomarkers reflecting NET formation.

## NETs

Immunothrombosis has been extensively described to contribute to the pathophysiology of severe COVID-19. Neutrophil extracellular traps (NETs) play a central role in immunothrombosis. NETs are neutrophil-derived expelled desoxyribonucleic acid (DNA) fibers decorated with antimicrobial granule proteins such as myeloperoxidase (MPO) and neutrophil elastase (NE) [1]. Increased plasma or serum levels of cell-free DNA, MPO/DNA complexes, NE/DNA complexes and citrullinated histone 3 (H3Cit) have been used as indicators for in vivo NET formation in various disease settings. The detection of a single NET component is considered rather unspecific. Cell-free DNA is least informative for determining NET formation, as extracellular DNA could be derived from any cell type undergoing cell death. Measuring MPO/DNA complexes or NE/DNA complexes in contrast is much more specific to quantify NET fragments. In addition, determining H3Cit levels allows to reveal whether the NET formation process was dependent on the enzyme peptidylarginine deiminase 4 (PAD4). PAD4 converts peptidylarginine to citrulline on the N-terminal of histone H3, thereby facilitating chromatin decondensation, which is a crucial step in NET formation [2]. It is recommended to measure NET markers in plasma rather than in serum because neutrophils could release NETs during clotting.

There are several studies showing that NETs can be detected and visualized in postmortem lung specimens from COVID-19 patients. However, here, we focus on the findings with soluble NET markers in plasma or serum from COVID-19 patients which could serve as diagnostic or prognositic biomarkers (Table 1).

**Table 1:** Collection of biomarkers described to be elevated in COVID-19 patients

Biomarker	Measured in				Associated with	
	Blood	Plasma	Serum	BALF	Severity	Thrombosis
*NETs*						
DNA		Ng et al. [10] Leppkes et al. [11] Zhang et al. [12]	Zuo et al. [3] Leppkes et al. [11]		Zuo et al. [3] Ng et al. [10] Leppkes et al. [11] Zhang et al. [12]	Zuo et al. [4]
his-DNA		Ouwendijk et al. [6]	Guéant et al. [13]			
MPO/DNA		Middleton et al. [8] Skendros et al. [7] Veras et al. [9] Leppkes et al. [11] Petito et al. [5] Ouwendijk et al. [6] Zhang et al. [12]	Zuo et al. [3] Guéant et al. [13]		Middleton et al. [8] Zuo et al. [3] Petito et al. [5] Ouwendijk et al. [6] Zhang et al. [12]	Zuo et al. [4] Petito et al. [5]
NE/DNA		Leppkes et al. [11]				
H3Cit		Ng et al. [10] Petito et al. [5]	Zuo et al. [3] Leppkes et al. [11]		Ng et al. [10] Leppkes et al. [11] Petito et al. [5]	Zuo et al. [4] Petito et al. [5]
*Complement*						
C3		Zhang et al. [12]			Zhang et al. [12]	
C5		Zhang et al. [12]			Zhang et al. [12]	
C5a		Carvelli et al. [25] Cugno et al. [26] Cugno et al. [27] Holter et al. [28]		Carvelli et al. [25]	Carvelli et al. [25]	
sC5b-9		Skendros et al. [7] Cugno et al. [26] Cugno et al. [27] Holter et al. [28]			Cugno et al. [26] Cugno et al. [27] Holter et al. [28]	
*Others*						
miRNA^a^	Li et al. [14]					
ACE2			Nagy et al. [16]	Garvin et al. [15]		
HMGB1			Chen et al. [18]		Chen et al. [18]	
PGRN			Rieder et al. [20]		Rieder et al. [20]	
CPN		Shi et al. [23] Silvin et al. [24]	Zuo et al. [4] Bauer et al. [21] Shi et al. [23] Luis et al. [22]		Bauer et al. [21] Shi et al. [23] Silvin et al. [24] Luis et al. [22]	Zuo et al. [4]
NE		Ng et al. [10]			Ng et al. [10]	
MMP-9		Petito et al.^5^				

^a^Certain miRNAs were upregulated and others were downregulated in COVID-19 patients

Zuo et al. [3] were the first to provide evidence for the occurrence of NETs in COVID-19 patients. Cell-free DNA, MPO/DNA complexes and H3Cit were shown to be increased in sera from 50 hospitalized patients with COVID-19 in comparison to 30 healthy controls. Notably, higher serum levels of cell-free DNA and MPO/DNA complexes were detected in more severe COVID-19 patients. Moreover, sera from COVID-19 patients induced NET formation in control neutrophils. The same group later complemented their initial findings by a further study showing that these three NET markers were not only elevated in COVID-19 patients, but also associated with a higher risk of developing thrombotic events [4].

Thrombotic complications were also linked to NETs in a study by Petito and colleagues [5]. Their study included 36 COVID-19 patients and 31 healthy controls. Venous thromboembolism was detected in 22% of COVID-19 patients. MPO/DNA complexes and H3Cit plasma levels were elevated in COVID-19 patients compared to healthy controls. Both NET markers correlated with disease severity and were associated with thrombotic events. Further, COVID-19 patients had increased plasma levels of matrix metalloproteinase 9 (MMP-9), an important enzyme for neutrophil extravasation and migration, but with no effect on clinical outcome.

In a study from Ouwendijk et al. [6] investigating 75 COVID-19 patients and 7 healthy controls, cell-free histone-DNA (his-DNA) and MPO/DNA complexes were increased in plasma from COVID-19 patients. MPO/DNA levels correlated with disease severity but were not associated with thrombosis.

Skendros et al. [7] shed light on the mechanism how NETs could promote thrombotic events in COVID-19 patients. 25 COVID-19 patients had elevated plasma levels of MPO/DNA complexes, tissue factor (TF) activity, and sC5b-9 as compared to 10 healthy individuals. They performed several in vitro assays using neutrophils and plasma from COVID-19 patients and concluded that NETs in concert with complement activation increase TF activity which may lead to thrombus formation.

Further studies investigated the utility of circulating NET markers as prognostic indicators for severity and clinical outcome in COVID-19.

Shortly after the first publication of NETs in COVID-19, Middleton and colleagues [8] confirmed that NETs are crucial players in the COVID-19 pathophysiology and highlighted their role in the development of acute respiratory distress syndrome in COVID-19. They studied 33 patients with COVID-19 and 17 healthy donors and found that the amount of MPO/DNA complexes in plasma was significantly higher in COVID-19 patients as compared to healthy controls. Strikingly, increased MPO/DNA complex plasma levels correlated with disease severity. Plasma from COVID-19 patients further induced NET formation in neutrophils isolated from healthy donors. In addition, they proposed that NET inhibitory peptides might be a therapeutic intervention strategy.

MPO/DNA complexes in plasma of COVID-19 patients were also studied by Veras et al. [9]. This group reported that levels of MPO/DNA complexes were increased in plasma of 32 patients with COVID-19 as compared to 21 healthy controls. Compared to all other studies presented here, it is the only study revealing that NET release could be induced by direct interaction of the virus SARS-CoV-2 with neutrophils.

The study of Ng et al. [10] enrolled 106 patients with moderate and severe disease. 30 healthy volunteers were included as controls. Cell-free DNA, H3Cit and NE were enhanced in plasma from COVID-19 patients and levels of NET markers were associated with respiratory support requirement and mortality.

Leppkes et al. [11] studied levels of cell-free DNA, MPO/DNA complexes, NE/DNA complexes in plasma and cell-free DNA, H3Cit in serum in a total of 70 subjects. All levels were enhanced in patients with COVID-19 compared to healthy controls. Patients with severe disease had higher levels of cell-free DNA and H3Cit than patients with a milder form.

135 COVID-19 patients and 25 healthy controls were enrolled in a study by Zhang et al. [12]. Cell-free DNA and MPO/DNA complexes were higher in plasma of patients with COVID-19 than in healthy controls and were associated with severity of COVID-19.

With a focus on the early response to SARS-CoV-2 infection, the study by Guéant et al. [13] assessed NET markers in an all-comers cohort involving 60 patients symptomatic with at least two typical COVID-19 symptoms and divided into SARS-CoV-2-positive (56.6%) and SARS-CoV-2-negative patients. 9 asymptomatic volunteers served as controls. Highest MPO/DNA serum levels were determined in the SARS-CoV-2-positive group followed by the SARS-CoV-2-negative group and by controls with significant differences between all groups. Serum levels of histone-DNA complexes were increased in both, SARS-CoV-2-positive and SARS-CoV-2-negative as compared to controls, without a significant difference between the two symptomatic groups.

Two phase 2 randomized controlled trials are currently recruiting patients to investigate the therapeutic potential of recombinant human deoxyribonuclease I (rhDNase I)—Pulmozyme—in patients with COVID-19 (NCT04402944 and NCT04432987), a drug that was shown to clear NETs and is part of routine treatment of patients with cystic fibrosis.

## miRNAs

In the peripheral blood of 10 patients with COVID-19, 35 miRNAs were upregulated and 38 miRNAs were downregulated as compared to blood samples of 4 healthy donors in an analysis performed by Li et al. [14]. Among the 10 most upregulated transcripts (hsa-miR-16-2-3P, hsa-miR-5695, hsa-miR-10399-3P, hsa-miR-6501-5P, hsa-miR-361-3P, hsa-miR-361-3p, hsa-miR-4659a-3p, hsa-miR-142-5p, hsa-miR-4685-3p, hsa-miR-454-5p, and hsa-miR-30c-5p), hsa-miR-16-2-3P was the one showing the clearest signal with a 1.6-fold upregulation.

## ACE2

Angiotensin-converting enzyme 2 (ACE2) is an entry receptor for SARS-CoV-2.

Garvin et al. [15] demonstrated upregulated ACE2 gene expression in bronchoalveolar lavage fluid (BALF) samples from COVID-19 patients.

A case report recently described the increase of serum ACE2 in a COVID-19 patient presenting with ARDS [16].

However, in a larger cohort of patients and at an earlier stage of infection, ACE2 levels in serum were not increased in SARS-CoV-2-positive patients as compared to well-matched SARS-CoV-2-negative control patients [17].

## HMGB1

Chen et al. [18] demonstrated that the chromatin protein and transcription regulator high mobility group box protein 1 (HMGB1) was elevated in sera of severe COVID-19 patients. Since HMGB1 was further shown to promote expression of SARS-CoV-2 entry receptor ACE2 in alveolar epithelial cells, targeting HMGB1 by pharmacological inhibition might be a potential treatment option for COVID-19 patients.

## Progranulin

Progranulin (PGRN) is predominantly expressed in epithelial cells, neurons and macrophages but is also found in other cell types. PGRN is involved in inflammation, wound healing and cell proliferation [19]. It was recently identified in a serum protein expression screen of emergency room all-comers with COVID-19-typical symptoms and positive versus negative SARS-CoV-2 status [20]. PGRN was upregulated in COVID-19 patients and associated with adverse outcomes and increased IL-6 expression. These findings need to be confirmed in a larger cohort including patients with severe COVID-19 ARDS, but the magnitude of the results make PGRN a promising serum marker.

## Calprotectin

Calprotectin (S100A8/S100A9) is an abundant protein complex in the cytosol of neutrophils. Some studies showed elevated calprotectin levels in sera [4, 21–23] from COVID-19 patients while others detected higher calprotectin levels in COVID-19 plasmas [23, 24]. Most of these studies could correlate high calprotectin concentrations with disease severity [21–24]. Only one group discovered that calprotectin serum levels in COVID-19 patients were associated with high thrombotic risk [4].

## Complement

The usefulness of complement factors (C3 [12], C5 [12], C5a [25–28], sC5b-9 [7, 26–28]) as biomarkers for COVID-19 has been suggested by several groups. All of the studies were performed with assays using plasma samples and demonstrated increased levels in COVID-19 patients. In parallel to plasma, Carvelli et al. [25] tested BALF samples. The association of elevated levels of complement factors with severe courses of COVID-19 could be verified by most of the groups [12, 25–28].

## Further biomarkers not yet backed by clinical data

Some molecules have been proposed as potential biomarkers and therapeutic targets, but have not yet been verified in COVID-19 patients.

One promising candidate is the transcription factor hypoxia-inducible factor 1-alpha (HIF-1α) which is a key regulator of oxygen homeostasis [29]. HIF-1α can modify the expression of molecules which mediate SARS-Cov-2 entrance such as angiotensin-converting enzyme 2 (ACE2) and transmembrane protease serine 2 (TMPRSS2). Experimental data also suggests that HIF-1α stabilization by reactive oxygen species (ROS) mediates production of proinflammatory cytokines and the formation of NETs. Consequently, HIF-1α could serve as a possible marker for the course of disease and a possible target in COVID-19 therapy.
